# A Linear Discriminant Analysis Model Based on the Changes of 7 Proteins in Plasma Predicts Response to Anlotinib Therapy in Advanced Non-Small Cell Lung Cancer Patients

**DOI:** 10.3389/fonc.2021.756902

**Published:** 2022-01-07

**Authors:** Fei Xu, Haiyan Xu, Zhiyi Wan, Guangjian Yang, Lu Yang, Xueying Wu, Jin Song, Yan Wang

**Affiliations:** ^1^ Department of Medical Oncology, National Cancer Center/National Clinical Research Center for Cancer/Cancer Hospital, Chinese Academy of Medical Sciences and Peking Union Medical College, Beijing, China; ^2^ Department of Comprehensive Oncology, National Cancer Center/National Clinical Research Center for Cancer/Cancer Hospital, Chinese Academy of Medical Sciences and Peking Union Medical College, Beijing, China; ^3^ Genecast Precision Medicine Technology Institute, Beijing, China; ^4^ Beijing Immupeutics Medicine Technology Limited, Beijing, China

**Keywords:** anlotinib, non-small cell lung cancer, linear discriminant analysis, predict, progression-free survival, protein

## Abstract

**Background:**

Anlotinib is a multi-targeted tyrosine kinase inhibitor mainly targeting angiogenesis signaling. The predictive marker of anlotinib’s efficacy remains elusive. This study was designed to explore the predictive marker of anlotinib in non-small cell lung cancer (NSCLC).

**Methods:**

We prospectively enrolled 52 advanced NSCLC patients who underwent at least one line of targeted therapy or chemotherapy between August 2018 and March 2020. Patients were divided into durable responders (DR) and non-durable responders (NDR) based on the median progression-free survival (PFS, 176 days). The Olink Immuno-Oncology panel (92 proteins) was used to explore the predictive protein biomarkers in plasma samples before treatment (baseline) and on the first treatment evaluation (paired).

**Results:**

At baseline, the response to anlotinib was not significantly associated with age, gender, smoke history, histology, oligo-metastases, *EGFR* mutations, and other clinical characteristics. The results of PFS-related protein biomarkers at baseline were all not satisfying. Then we assessed the changes of 92 proteins levels in plasma on the first treatment evaluation. We obtained a Linear discriminant analysis (LDA) model based on 7 proteins, with an accuracy of 100% in the original data and an accuracy of 89.2% in cross validation. The 7 proteins were CD70, MIC-A/B, LAG3, CAIX, PDCD1, MMP12, and PD-L2. Multivariate Cox analysis further showed that the changes of CD70 (HR 25.48; 95% CI, 4.90–132.41, P=0.000) and MIC-A/B (HR 15.04; 95% CI, 3.81–59.36, P=0.000) in plasma were the most significant prognostic factors for PFS.

**Conclusion:**

We reported herein a LDA model based on the changes of 7 proteins levels in plasma before and after treatment, which could predict anlotinib responders among advanced NSCLC patients with an accuracy of 100%. Further studies are warranted to verify the prediction performance of the LDA model.

## Introduction

Non-small cell lung cancer (NSCLC) is the leading cause of cancer mortality in the world and in China (1, 2). Although the treatment of NSCLC has made considerable progression with the development of precision medicine, effective three or later lines of therapy for advanced NSCLC is still scarce. Anlotinib, a multitargeted antiangiogenic drug, has been used in third-line therapy for refractory advanced NSCLC in China (3). In the ALTER0303 trial, anlotinib significantly prolonged overall survival (OS) and progression-free survival (PFS) (3). Although the disease control rate (DCR) is 80.95%, the objective response rate (ORR) is only 9.18% (3). Therefore, screening predictors of response to anlotinib is an urgent clinical need.

To date, intensive efforts have been performed to screen the predictors of response to anlotinib in lung cancer. These predictors include circulating tumor DNA (ctDNA) (4), expression of *KLK5* and *L1CAM* (5), nutritional indicators (6), plasma CCL2 levels (7), plasma metabolites (8), prognostic nutritional index (9), absolute neutrophil count (ANC) (10), and etc. Unfortunately, due to the complexity of the angiogenesis pathway, effective biomarkers of response to anlotinib still require further exploration. For example, the area under the curve (AUC) of PFS and OS response prediction based on the tumor mutation index of ctDNA is only 0.77 and 0.73, respectively (4). ANC has a relatively high discriminatory ability to predict 10-month survival with an area under the curve of 0.729 (10).

Studies reveal that there are many interactions between the tumor immune microenvironment and angiogenesis. The overexpression of VEGF can directly suppress immune effector cells and activate immunosuppressive cells, while activated immunosuppressive cells further promote abnormal angiogenesis by secreting various factors (11, 12). For instance, tumor-associated macrophages and neutrophils promote angiogenesis by secreting various chemokines and proangiogenic factors including CXCR-2,4,12, CXCL3,4,8,9,10, CCL2-5, VEGF, TNFa, and IL8 (13). Anlotinib can increase infiltration of the innate immune cells and modulate the tumor microenvironment (14). Clinical studies of combining antiangiogenic agents and immune checkpoint inhibitors have been reported in a variety of solid tumors (15–17). Recently, phase 1b study of sintilimab plus anlotinib as first-line therapy for advanced NSCLC patients has been performed (18).

Plasma-based protein biomarker tests are routinely used for predicting prognosis and monitoring efficacy of treatment. Given the key role of cytokines in the interaction of angiogenesis and tumor immune microenvironment, we investigated the levels of 92 immuno-oncology related proteins in plasma with the aim of identifying effective predictors of response to anlotinib in NSCLC patients.

## Methods

### Patients and Samples

Patients and samples were collected from the Chinese clinical trial (ChiCTR1800017585). We investigated 52 advanced NSCLC patients who underwent at least one line of targeted therapy or chemotherapy at Cancer Hospital, Chinese Academy of Medical Sciences & Peking Union Medical College (Beijing, China) between August 2018 and March 2020. All patients were administered with anlotinib as a second-line therapy or over second-line therapy with a dosage of 12 mg/day in 2/1 week cycles. Anlotinib therapy was terminated if intolerable toxicity or disease progression occurred. Response to anlotinib was evaluated by oncologists and radiologists based on clinical and radiological information. Durable responders (DR) were defined as patients with PFS ≥ 176 days (median PFS), while non-durable responders (NDR) were defined as patients with PFS < 176 days. This study was conducted in accordance with the Declaration of Helsinki and approved by the ethical committee of Cancer Hospital, Chinese Academy of Medical Sciences & Peking Union Medical College (approval number: No. 18-116/1694). All patients had signed the informed consent.

Blood samples were collected before treatment (baseline) and on the first treatment evaluation (paired). The blood samples were centrifuged at 2000–2500 g for 10 min and the plasma samples were then stored at −80°C until assayed.

### Protein Profiling

Protein concentrations in plasma were measured using the Immuno-Oncology panel (Olink Biosciences, Uppsala, Sweden) including 92 protein biomarkers according to the manufacturer’s instructions and as described before (19). The protein quantifications were determined as the relative quantification using the normalized protein expression and presented as log2-normalized. The list of all protein biomarkers is provided in **Table S1**. A total of 43 plasma samples at baseline and 37 paired plasma samples after the second treatment cycle were successfully detected and used for further analysis. The change of protein levels in the plasma during treatment was compared between samples after the second treatment cycle and the corresponding sample at baseline using a paired two-sided t test, and quantified with the log2-fold change (log2-FC).

### Statistical Analysis

All statistical analyses were completed using R version 3.6.3 or SPSS v25. Patient characteristics at baseline were compared using Fisher’s exact test for categorical variables, and Mann−Whitney U test for continuous variables. PFS and OS were analyzed using the Kaplan-Meier method. Receiver operating characteristics (ROC) curve was calculated to evaluate the discriminatory ability for each protein at baseline. The association between factors and PFS was analyzed using univariate or multivariate Cox proportional hazards model. Statistical tests were two-sided, and p < 0.05 was considered significant. Principal component analysis (PCA) and hierarchical clustering were performed using the PAST software. Hierarchical clustering was applied based on Euclidean distance. Linear discriminant analysis (LDA) and Leave-One-Out cross validation were performed using SPSS v25.

## Results

### Patient Characteristics

**Figure 1:**
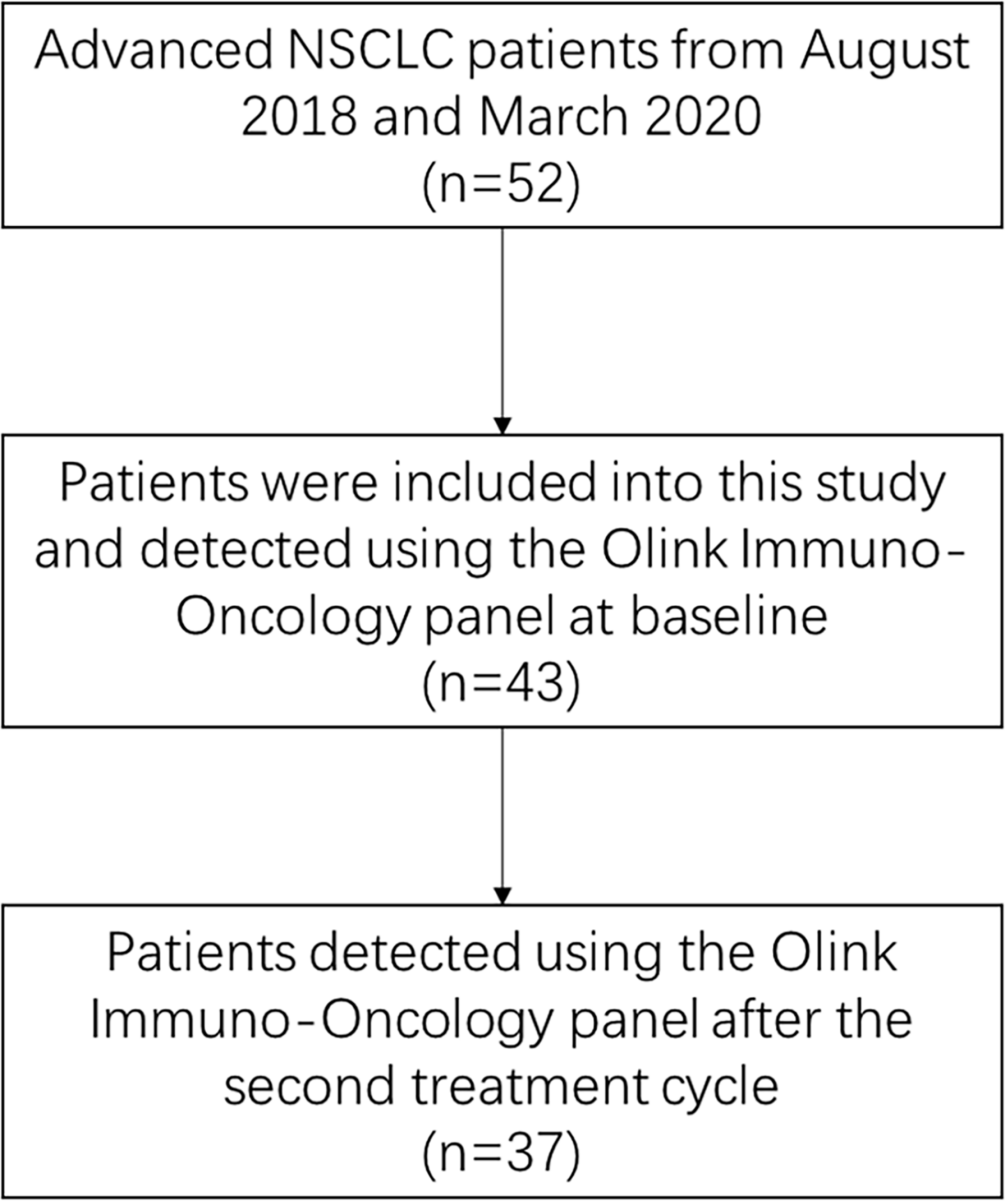
Flow chart of the study.

The flow chart of patients is shown in **Figure 1**. After quality control of patients’ plasma samples, a total of 43 patients were enrolled and detected at baseline. The average age at diagnosis was 60.8 ± 8.7 years and 48.84% (21/43) were male. Central nervous system (CNS) metastases and oligo-metastases were present in 18.60% (8/43) and 48.84% (21/43) patients, respectively. The objective response rate (ORR) was 6.98% (3/43), all based on achieving partial response. In addition, 83.72% (3/43) patients achieved stable disease, and 9.30% (4/43) had progressive disease. The median PFS and OS were 176 days and 311 days, respectively. Based on the median PFS, all patients were divided into DR group (PFS≥176 days) and NDR group (PFS < 176 days). DR and NDR group did not have differences in all clinical characteristics at baseline, including histology and *EGFR* mutations (**Table S2**). Univariate analysis was then performed using COX proportional hazards model, only neutrophil-to-lymphocyte ratio (NLR, HR 1.42; 95% CI, 1.11–1.81; P=0.005) was significantly associated with PFS at baseline (**Figure S1**).

### Protein Plasma Levels at Baseline

To explore the relationship between protein biomarkers in plasma and clinical response, we simultaneously detected 92 proteins levels in plasma at baseline and after the second treatment cycle using highly sensitive proximity extension assay (PEA) technology (19).

At baseline, a total of 43 plasma samples were detected and analyzed (**Table S3**). Firstly, ROC showed that the area under the curve (AUC) of each protein was less than 0.7 (data not shown). Levels of 92 proteins were then compared between DR group and NDR group using unsupervised hierarchical clustering and PCA. However, these two groups cannot be effectively distinguished (**Figure S2**). LDA cannot be performed due to too many variables. Therefore, we then analyzed the association between each protein level and PFS by Cox univariate proportional hazards models. The results showed that 4 proteins were significantly associated with PFS, including CD8A (HR 0.42; 95% CI, 0.23–0.77; P=0.005), MUC.16 (HR 1.23; 95% CI (1.02–1.48; P<0.033), IL5 (HR 1.63; 95% CI, 1.07–2.50; P=0.034), and KIR3DL1 (HR 1.53; 95% CI, 1.04–2.27; P=0.041) (**Figure S1**). Lastly, unsupervised hierarchical clustering, PCA, and LDA were further performed based on 5 factors significantly associated with PFS including NLR, CD8A, MUC.16, IL5, and KIR3DL1. Unfortunately, none of these methods can effectively distinguish between DR and NDR groups, although LDA achieved 65% accuracy (data not shown).

### Change in Protein Plasma Levels Is Associated With Anlotinib Treatment

Before treatment, levels of 92 proteins in plasma cannot effectively predict the efficacy of anlotinib. Therefore, we tried to analyze the correlation between the changes in 92 proteins on the first treatment evaluation and the efficacy of anlotinib. The change of each protein during treatment was represented as log2 fold change (log2 FC). After the second treatment cycle, which was the time of the first treatment evaluation, a total of 37 paired plasma samples were analyzed (**Table S4**).

**Figure 2:**
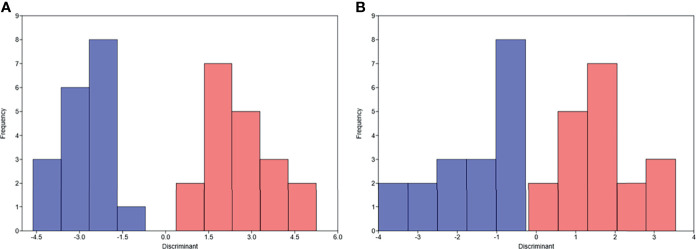
Linear discriminant analysis (LDA) of progression-free survival based on the changes of proteins in plasma after the second treatment cycle. **(A)** LDA based on the changes of 25 proteins. **(B)** LDA based on the changes of 7 proteins. Pink, durable responders; Blue, non-durable responders.

**Figure 3:**
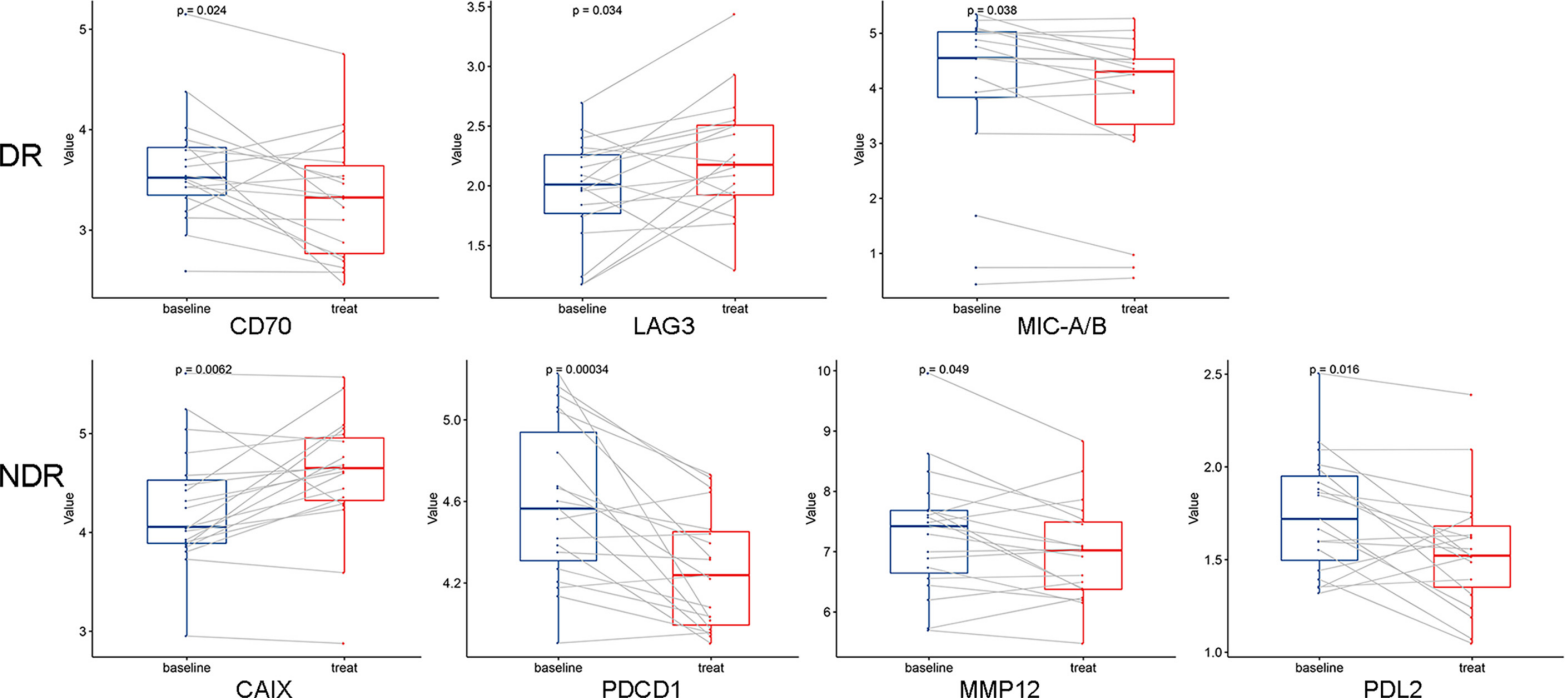
Dynamic changes of 7 proteins used in the linear discriminant analysis prediction model. A paired two-sided t test was used, a=0.05. DR, durable responders; NDR, non-durable responders.

**Figure 4:**
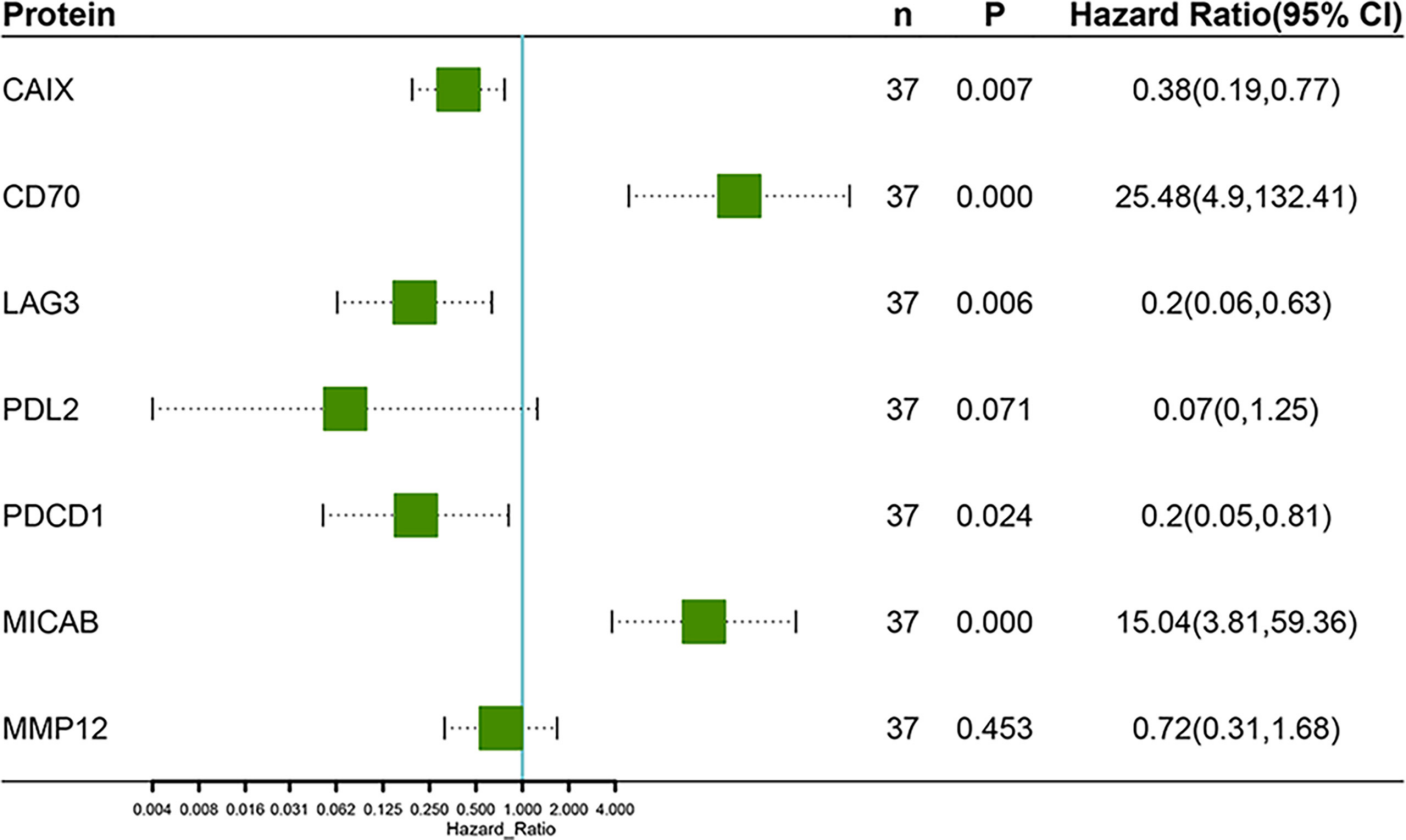
Multivariate Cox regression analysis of progression-free survival based on the changes of 7 proteins after the second treatment cycle.

Unsupervised hierarchical clustering and PCA based on the changes of the 92 proteins cannot effectively distinguish between DR group and NDR group (data not shown). Then, we focused on the proteins that have changed significantly during treatment. There were 13 and 21 proteins that changed significantly during treatment in DR group and NDR group, respectively. There were 25 proteins in the union set of the two groups. Then, we performed unsupervised hierarchical clustering, PCA, and LDA using these 25 proteins. The results of unsupervised hierarchical clustering and PCA were also not ideal (data not shown). However, LDA model achieved 100% accuracy (**Figure 2A**). Through iterative subtraction analysis, we screened out 7 proteins for LDA, with an accuracy of 100% (**Figure 2B**). Leave-One-Out cross validation further showed that the model had 89.2% accuracy (**Table 1**). The 7 proteins were CD70, MIC-A/B, and LAG3 in DR group and CAIX, PDCD1, MMP12, and PD-L2 in NDR group (**Figure 3**). In DR group, the plasma levels of CD70 and MIC-A/B were decreased significantly after the second treatment cycle, while the level of LAG3 increased significantly (**Figure 3**). Multivariate Cox analysis based on the 7 proteins was further performed. The results showed that the decreases of CD70 (HR 25.48; 95% CI, 4.90–132.41, P=0.000) and MIC-A/B (HR 15.04; 95% CI, 3.81–59.36, P=0.000) in plasma after treatment were the most significant prognostic factors for PFS (**Figure 4**).

**Table 1:** Leave-one-out cross validation of the linear discriminant analysis (LDA) model.

		LDA prediction		Total	Accuracy
		NDR	DR		
Group	NDR	16	2	18	89.2%
	DR	2	17	19

DR, durable responders; NDR, non-durable responders.

## Discussion

It could be difficult for previous studies to find the predictive markers of antiangiogenic drugs since they could not be used alone in non-small cell lung cancer (20). Anlotinib is an antiangiogenic drug that approved for third-line or further-line treatment of advanced NSCLC. This has enabled us to explore the biomarkers of antiantiogenic drug alone. However, the effective biomarkers for predicting the efficacy of anlotinib remain further exploration, although studies have revealed some potential biomarkers for anlotinib response stratification (4–8, 10, 21). The evolution of cancer is influenced by intricate interactions between tumor cells and host immune cells. Vascular endothelial cells and immune cells are important components in tumor-associated inflammation, and inflammatory response is an important factor in the progression of tumor (22). The products of inflammatory processes including various cytokines and chemokines are considered to be potential biomarkers for predicting treatment efficacy (23). Besides, apart from subgroup analysis in ALTER0303, recent studies have also shown that anlotinib could have efficacy in treating driver-gene positive lung tumors (24, 25), and basic research showed that there was a potential link between EGFR/KRAS mutation and VEGF expression (26). A broader biomarker was to be developed which could be applied to patients with different genetic background.

Here, we sought to identify biomarkers of response to anlotinib in advanced NSCLC patients using the Olink Immuno-Oncology panel in plasma samples. The Immuno-Oncology panel is a high-throughput, multiplex immunoassay enabling analysis of 92 protein biomarkers (27). These proteins are involved in processes such as promotion and inhibition of tumor immunity, chemotaxis, vascular and tissue remodeling, apoptosis, metabolism, and autophagy.

In this study, a total of 43 NSCLC patients’ samples were detected at baseline. The median PFS and OS is 176 days and 311 days, respectively, which were in line with the report of ALTER0303 trial and some other studies (3, 10, 28). The PFS is a reliable indicator that directly reflects the treatment effect. Therefore, patients were divided into the DR group and NDR group based on the median PFS. There was no significant difference in the response to anlotinib (both PFS and OS) between SCC and ADC, which was also consistent with the results of ALTER0303 trial (3). In addition, no significant correlation was observed between the response to anlotinib and *EGFR* mutation status. Univariate Cox analysis was performed to assess the potential prognostic value of clinical characteristics. Only NLR was significantly associated with PFS.

Before treatment, the AUC of ROC curve for each protein did not exceed 0.7. We then used clustering analysis, PCA, and LDA to predict PFS based on all 92 proteins and/or significant correlated proteins obtained from Cox analysis. However, the results were all not effective enough. Then, we further assessed the potential prognostic value of the changes of 92 proteins levels in plasma after the second treatment cycle. After attempting various analytical methods, we finally obtained a LDA model based on 7 proteins, with an accuracy of 100% in the original data and an accuracy of 89.2% in cross validation. This performance is much better than previous reports (4–6, 10). The 7 proteins included CD70, MIC-A/B, LAG3, MMP12, PD-L2, CAIX, and PDCD1. Multivariate Cox analysis further showed that CD70 and MIC-A/B were independent prognostic factors for PFS.

CD70 is a ligand for CD27 and expressed on highly activated lymphocytes. CD27-CD70-mediated T cell co-stimulation was also important for vasculogenesis, arteriogenesis, and angiogenesis (29). MIC-A/B are ligands for the NKG2D receptor. These proteins are broadly recognized by NK cells, NKT cells, and most of the subtypes of T cells (30). LAG3 binds to HLA class-II antigens and is an immune checkpoint receptor. It is expressed on multiple cell types and involved in lymphocyte activation and homeostasis (31). PDCD1, an important immune checkpoint, plays a significant role in controlling and resolving immune responses (32). MMP-12 is secreted by pro-inflammatory macrophages that targets endoglin in human macrophages and endothelial cells (33). CAIX is one of the best cellular biomarkers of hypoxia and plays a crucial role in growth, migration, invasion, and metastasis of tumors (34). Overall, these 7 proteins were mainly involved in co-stimulatory molecule, hypoxia, inhibitory checkpoint, and metalloproteinase. Interestingly, some angiogenesis-related proteins (such as VEGFR-2, VEGFA, and TIE2) were significantly changed during treatment in both DR and NDR group, but no correlation was observed between change of these proteins and PFS.

This study not only provide a possible response biomarker for anlotinib treatment in NSCLC, but might give a hint for upcoming treatment strategy combo in advanced NSCLC patients. Up till now, anlotinib plus immunotherapy has been reviewed in later lines (35–37) and took a chance in front-lines in clinical trials (18). One limitation of this study is the lack of a validation group due to the small number of patients, which may affect the accuracy of the LDA model based on the changes of 7 proteins in the real world. More NSCLC patients are needed for verifying the prediction performance of the LDA model based on 7 proteins.

In conclusion, we reported herein a LDA model based on the changes of 7 proteins levels in plasma after the second treatment cycle, which could predict anlotinib responders among refractory advanced NSCLC patients with an accuracy of 100% in the original data and an accuracy of 89.2% in cross validation. In addition, levels of the 92 proteins in plasma cannot effectively predict anlotinib responders at baseline. These results may have a potential clinical implication for guiding treatment of anlotinib therapy in advanced NSCLC.

## Data Availability Statement

The original contributions presented in the study are included in the article/**Supplementary Material**. Further inquiries can be directed to the corresponding authors.

## Ethics Statement

The studies involving human participants were reviewed and approved by Cancer Hospital, Chinese Academy of Medical Sciences & Peking Union Medical College. Written informed consent to participate in this study was provided by the participants’ legal guardian/next of kin.

## Author Contributions

Conception and design: YW and JS. Provision of study materials or patients: FX, HX, and YW. Collection and assembly of data: FX, HX, GY, and LY. Data analysis and interpretation: FX, HX, ZW, and XW. Manuscript writing: All authors; All authors contributed to the article and approved the submitted version.

## Conflict of Interest

Author ZW and XW were employed by the company Genecast Precision Medicine Technology Institute, Beijing, China. Author JS was employed by the company Beijing Immupeutics Medicine Technology Limited, Beijing, China.

The remaining authors declare that the research was conducted in the absence of any commercial or financial relationships that could be construed as a potential conflict of interest.

## Publisher’s Note

All claims expressed in this article are solely those of the authors and do not necessarily represent those of their affiliated organizations, or those of the publisher, the editors and the reviewers. Any product that may be evaluated in this article, or claim that may be made by its manufacturer, is not guaranteed or endorsed by the publisher.
