# Supplementary material for: A Linear Discriminant Analysis Model Based on the Changes of 7 Proteins in Plasma Predicts Response to Anlotinib Therapy in Advanced Non-Small Cell Lung Cancer Patients
Source: Front Oncol. 2022 Jan 7;11:756902. doi: 10.3389/fonc.2021.756902 (PMC8777128; doi:10.3389/fonc.2021.756902)
Supplement: Supplementary file 1 [file DataSheet_1.docx]

Supplementary Material

## Supplementary Figures


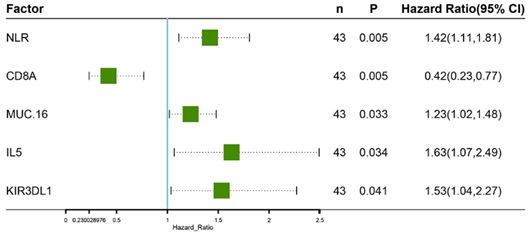


**Supplementary Figure 1.** Univariate Cox regression analysis of progression-free survival at baseline. NLR, Neutrophil-to-lymphocyte ratio.


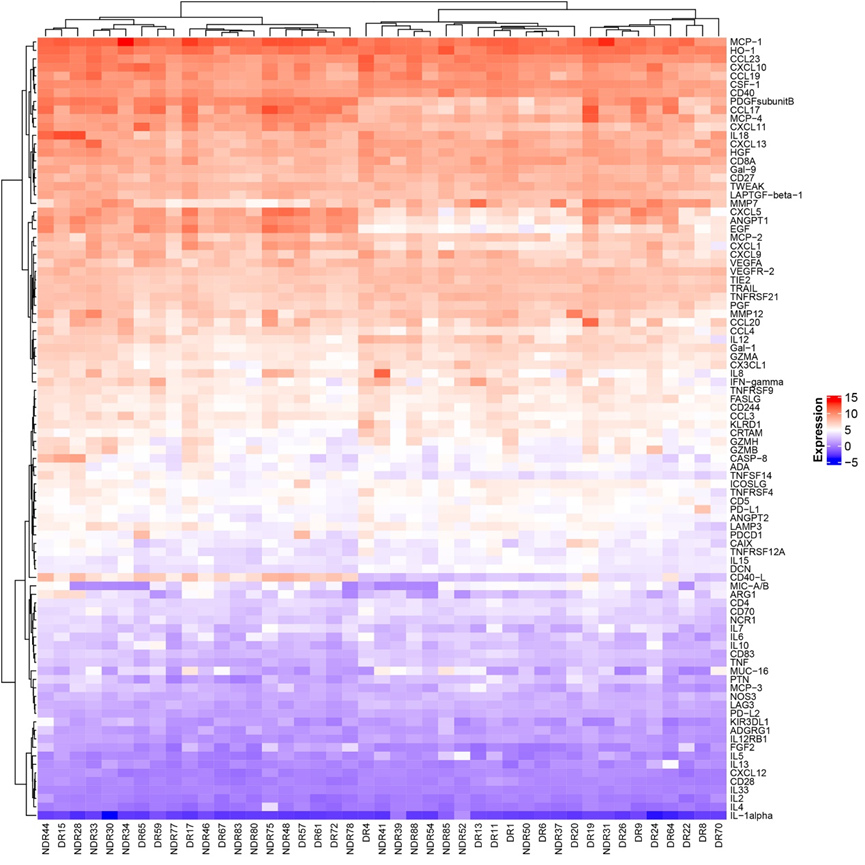


**Supplementary Figure 2.** Hierarchical clustering heatmap of 92 proteins levels in plasma at baseline. DR, durable responders; NDR, non durable responders.

## Supplementary Tables

**Supplementary Table 1.** The protein list of the Immuno-Oncology panel.

| ADA | CD27 | CXCL11 | HGF | IL5 | MMP12 | TNFRSF21 |
| --- | --- | --- | --- | --- | --- | --- |
| ADGRG1 | CD28 | CXCL12 | HO-1 | IL6 | MMP7 | TNFRSF4 |
| ANGPT1 | CD4 | CXCL13 | ICOSLG | IL7 | MUC-16 | TNFRSF9 |
| ANGPT2 | CD40 | CXCL5 | IFN-gamma | IL8 | NCR1 | TNFSF14 |
| ARG1 | CD40-L | CXCL9 | IL10 | KIR3DL1 | NOS3 | TRAIL |
| CAIX | CD5 | DCN | IL12 | KLRD1 | PDCD1 | TWEAK |
| CASP-8 | CD70 | EGF | IL12RB1 | LAG3 | PDGFsubunitB | VEGFA |
| CCL17 | CD83 | FASLG | IL13 | LAMP3 | PD-L1 | VEGFR-2 |
| CCL19 | CD8A | FGF2 | IL15 | LAPTGF-beta-1 | PD-L2 |  |
| CCL20 | CRTAM | Gal-1 | IL18 | MCP-1 | PGF |  |
| CCL23 | CSF-1 | Gal-9 | IL-1alpha | MCP-2 | PTN |  |
| CCL3 | CX3CL1 | GZMA | IL2 | MCP-3 | TIE2 |  |
| CCL4 | CXCL1 | GZMB | IL33 | MCP-4 | TNF |  |
| CD244 | CXCL10 | GZMH | IL4 | MIC-A/B | TNFRSF12A | |

**Supplementary Table 2.** Clinicopathological characteristics of NSCLC patients (n=43).

| Clinicopathological features | NDR | DR | p value |
| --- | --- | --- | --- |
| Gender |  |  |  |
| Male | 10 | 11 | 1 |
| Female | 11 | 11 |  |
| Age (years) |  |  | 1 |
| ≥ 65 | 9 | 9 |  |
| < 65 | 12 | 13 |  |
| Smoke |  |  | 0.763 |
| No | 11 | 13 |  |
| Yes | 10 | 9 |  |
| Histology |  |  | 0.457 |
| ADC | 18 | 16 |  |
| SCC | 3 | 6 |  |
| *EGFR* mutations |  |  | 0.223 |
| Positive | 12 | 7 |  |
| Negative | 10 | 14 |  |
| Cohort |  |  | 0.535 |
| A | 3 | 6 |  |
| B | 7 | 8 |  |
| C | 11 | 8 |  |
| Pre_angio |  |  | 0.547 |
| Yes | 11 | 9 |  |
| No | 10 | 13 |  |
| Oligometastasis |  |  | 0.763 |
| M | 11 | 10 |  |
| S | 10 | 12 |  |
| CNS metastasis |  |  | 0.698 |
| Yes | 3 | 5 |  |
| No | 18 | 17 |  |
| HTN |  |  | 1 |
| Yes | 9 | 7 |  |
| No | 14 | 13 |  |
| NLR (median [IQR]) | 3.50 [2.05, 4.31] | 2.57 [1.67, 3.02] | 0.099 |
| Hb (median [IQR]) | 132.50 [119.00, 145.00] | 139.00 [108.00, 145.00] | 0.99 |
| PLT (mean (SD)) | 225.82 (85.00) | 237.00 (67.57) | 0.637 |

P-values are obtained with Fisher or Wilcoxon test.

DR, Durable responders; NDR, No durable responders; ADC, Adenocarcinoma; SCC, Squamous cell carcinoma; A, SCC patients; B, ADC patients without driver gene mutation; C, ADC patients with driver gene mutation; CNS, Central nervous system; SD, Standard deviation; IQR, Interquartile range; NLR, Neutrophil-to-lymphocyte ratio; PLT, Platelet; Hb, Hemoglobin; HTN, hypertension.
